# Antimicrobial Peptides and Ectosymbiotic Relationships: Involvement of a Novel Type IIa Crustin in the Life Cycle of a Deep-Sea Vent Shrimp

**DOI:** 10.3389/fimmu.2020.01511

**Published:** 2020-07-13

**Authors:** Simon Le Bloa, Céline Boidin-Wichlacz, Valérie Cueff-Gauchard, Rafael Diego Rosa, Virginie Cuvillier-Hot, Lucile Durand, Pierre Methou, Florence Pradillon, Marie-Anne Cambon-Bonavita, Aurélie Tasiemski

**Affiliations:** ^1^Ifremer, Univ. Brest, CNRS, Laboratoire de Microbiologie des Environnements Extrêmes (LM2E), Plouzané, France; ^2^Univ. Lille, CNRS, Inserm, CHU Lille, Institut Pasteur de Lille, U1019 – UMR 9017 - CIIL - Center for Infection and Immunity of Lille, Lille, France; ^3^Univ. Lille, CNRS, UMR 8198 - Evo-Eco-Paleo, Lille, France; ^4^Laboratory of Immunology Applied to Aquaculture, Department of Cell Biology, Embryology and Genetics, Federal University of Santa Catarina, Florianópolis, Brazil; ^5^Ifremer, Laboratoire Environnement Profond (REM/EEP/LEP), Plouzané, France

**Keywords:** extreme, hydrothermal, symbiosis, host-microbe interaction, invertebrate immunity, crustacean

## Abstract

The symbiotic shrimp *Rimicaris exoculata* dominates the macrofauna inhabiting the active smokers of the deep-sea mid Atlantic ridge vent fields. We investigated the nature of the host mechanisms controlling the vital and highly specialized ectosymbiotic community confined into its cephalothoracic cavity. *R. exoculata* belongs to the Pleocyemata, crustacean brooding eggs, usually producing Type I crustins. Unexpectedly, a novel anti-Gram-positive type II crustin was molecularly identified in *R. exoculata*. Re-crustin is mainly produced by the appendages and the inner surfaces of the cephalothoracic cavity, embedding target epibionts. Symbiosis acquisition and regulating mechanisms are still poorly understood. Yet, symbiotic communities were identified at different steps of the life cycle such as brooding stage, juvenile recruitment and molt cycle, all of which may be crucial for symbiotic acquisition and control. Here, we show a spatio-temporal correlation between the production of Re-crustin and the main ectosymbiosis-related life-cycle events. Overall, our results highlight (i) a novel and unusual AMP sequence from an extremophile organism and (ii) the potential role of AMPs in the establishment of vital ectosymbiosis along the life cycle of deep-sea invertebrates.

## Introduction

In marine habitats such as in deep-sea hydrothermal ecosystems, bacterial associations with invertebrates are well-described (1). The important animal biomass observed around hydrothermal vents is based on the existence of dense chemosynthetic prokaryotic communities (2, 3). Among these communities, a large number forms highly specialized symbiotic associations with metazoan hosts. These relationships are now quite well-studied, e.g., as in the case of the bacterial community inhabiting the cephalothoracic cavity of the shrimp *Rimicaris exoculata* (4–7). By contrast, understanding the mechanisms by which hosts selectively recruit bacteria for long-term (core) or short-term (flexible) specific relationships is still a considerable challenge (8). To date, most of the existing literature has focused on the role of immune receptors (lectins, PGRPs, FREPs, TLR, SRCR) in marine symbiotic associations (9–11). A given immune receptor recognizes some families of microbes (bacteria, fungi, viruses) on the basis of motifs of recognition called Microbe Associated Molecular Patterns (MAMPs) commonly exposed on the membrane of friend and foe microorganisms (12). However, other selective and specific host processes are required to discriminate between pathogenic or mutualistic microbes in order to selectively kill or tolerate them. Amongst the few other immune substances known to be involved in host-symbiont associations, host defense antimicrobial peptides (AMPs) represent promising actors (13–18). AMPs are chemical components that take part in both the internal and external immune defenses (i.e., they can be secreted in the outer parts of the body), thus playing functions in the control/establishment of ectosymbiosis as described for the hydrothermal worm *Alvinella pompejana* (19–21). From an evolutionary perspective, the adaptive diversification of AMPs at the interspecific and intraspecific levels makes them of particular interest to decipher the immune mechanisms driving bacteria-specific and environment-dependent symbioses (22–24).

The Pleocyemata shrimp *R. exoculata* dominates the fauna at several hydrothermal vent sites of the Mid-Atlantic Ridge (MAR) (25, 26). This deep-sea crustacean thrives in such hostile habitats through an association with two distinct ectosymbiotic microbial communities. One housed in its gut (27–29) and the other in its enlarged cephalothoracic cavity (4, 5, 7, 30–34). Previous studies have suggested and then demonstrated the chemotrophic role of the symbionts that colonize the cephalothoracic cavity (6, 35–37). This specialized ectosymbiosis composed of few specific bacterial lineages, mainly proteobacteria and *Campylobacterota* (previously *Epsilonproteobacteria*) (38) is confined to the internal faces of the lateral carapace (branchiostegites) and the mouthparts (scaphognathites) of the cephalothorax cavity, but not of the gills (5, 31, 32). While the gut symbiotic community harbors proteobacteria and *Campylobacterota*, other symbionts have been found in the digestive system (stomach and digestive tracts, respectively), such as Mollicutes or Deferribacteres (27–29) evoking an organ-dependent mode of selection of the symbionts by the host. Recently, ectosymbionts have also been described on eggs along their development (P. Methou, personal communication). Interestingly, every 10 days, the microbial community of the cephalothoracic cavity, but not of the gut, is eliminated during the molt of the adult and ectosymbionts rapidly re-colonize the host cephalothoracic cavity (39, 40). This re-colonization process is strictly similar for each individual and strictly located on the same area of mouthparts and branchiostegites, suggesting a tight selection of the bacteria by the host (5, 39). However, the immune mechanisms involved in this association remain mostly unknown. Only the recent work by Liu and his colleagues, has characterized the potential immune role of a C-type lectin highly expressed in the scaphognathites, which has a broad nonself-recognition spectra and could agglutinate some of the cephalothoracic symbionts (10, 11).

The immune system of crustaceans is based on cellular and humoral responses involving, among other substances, the production of AMPs (41). Several classes of both gene-encoded and non-ribosomally synthetized AMPs have been identified and characterized in major commercial species of decapod crustaceans (42, 43). To our knowledge, despite a particularly well-described role of these molecules in the immune response of crustaceans against pathogens (41), no studies have ever been conducted on their involvement in mutualistic symbiosis.

Crustins form a diverse and multigenic family of AMPs found in virtually all crustacean groups and in some hymenopteran insects (44). They are mainly active against Gram-positive bacteria, but their unique feature is the presence of a C-terminal whey acidic protein (WAP) domain, a conserved cysteine-rich motif (four-disulfide core or 4DSC) that exhibits antiprotease activities (45). Crustins are divided into four groups (Types I to IV) according to the presence/absence of two N-terminal structural domains: the glycine-rich and the cysteine-rich regions (43). Type I crustins contain an N-terminal cysteine-rich region (with four conserved cysteine residues) followed by the typical C-terminal WAP domain. In addition to the cysteine-rich region, Type II crustins also harbor a highly hydrophobic glycine-rich region at the N-terminus. Comparatively, crustin members from Types III and IV are composed of one and two WAP domains, respectively, and are devoid of any other domains. Interestingly, while Type I crustins are widely distributed across decapod crustaceans (Pleocyemata and Dendrobranchiata), Type II crustins (Sub-Types IIa and IIb) are mainly present in penaeid shrimps (Dendrobranchiata) (46).

In this study, we explored the sequence conservation of a novel glycine-rich crustin member, Re-crustin, produced by the extremophile Pleocyemata shrimp, *R. exoculata*. Then, we investigated expression patterns in different host tissues and throughout its life cycle in order to identify possible correlations with the main symbiosis related events taking place at different life stages of this vent shrimp. These events include embryonic development (5, 47), juvenile settlement into adult habitats (P. Methou, personal communication), and through the molt cycle involving re-establishment of the symbiotic community after each molting event (39).

## Materials and Methods

### Specimen Collection

**Figure 1:**
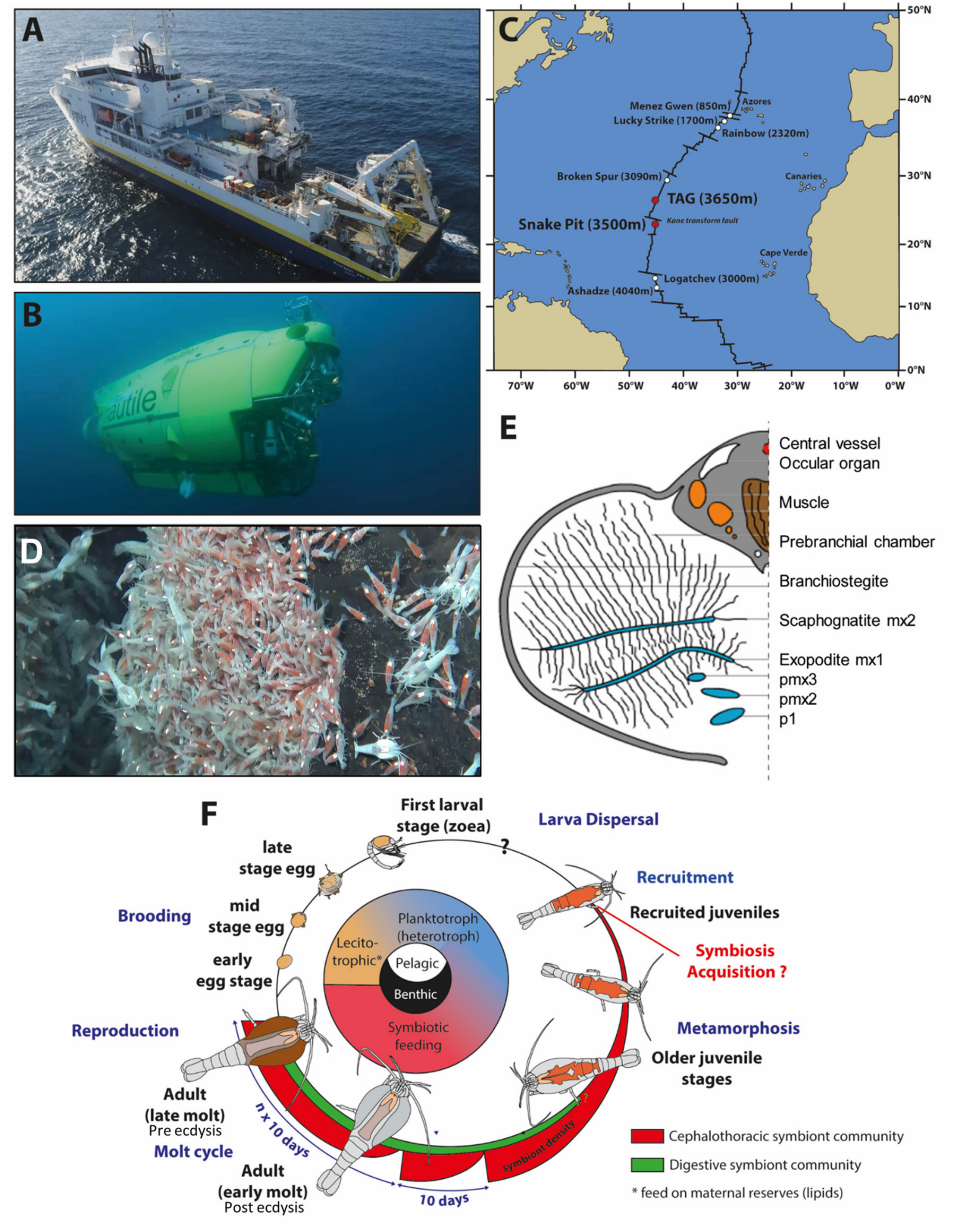
*Rimicaris exoculata* sampling and summary of its symbiotic relationships through its life cycle **(A)**. Research vessel (R/V) “*Pourquoi pas*?” and **(B)** HOV Nautile used for sampling. **(C)** Location of the two hydrothermal vent sites presently studied along the Mid-Atlantic Ridge. **(D)** Suction sampling of the shrimps at Snake Pit with the human operated submersible Nautile during the oceanographic cruise BICOSE2018. **(E)** *Rimicaris exoculata* cephalothoracic chamber [modified from Segonzac et al. (26)]. **(F)** Life cycle of *Rimicaris exoculata*. Inspired from the figure of Laming et al. (48) copyright BICOSE2-Nautile@Ifremer.

*Rimicaris exoculata* were collected at two MAR hydrothermal vent fields, TAG (26°08' N; −3,640 m) and Snake Pit (23°23' N; −3,480 m), with the Research Vessel (R/V) *Pourquoi pas?* using the suction sampler of the remotely operated vehicle (ROV) Victor 6,000 and the human operated submersible Nautile during the oceanographic cruises BICOSE2014 (https://doi.org/10.17600/14000100) and BICOSE2 2018 (http://doi.org/10.17600/18000004) (Figures 1A–C). The isobaric collection device PERISCOP (49) was used to collect shrimps at different life stages (several females with early or late eggs, recruited juveniles collected within adults' aggregates (Figure 1D), and adults at different molting stages). They were dissected aboard, and pieces were either flash frozen in liquid nitrogen before being kept at −80°C (with or without Trizol ReagentTM, Invitrogen) or were kept straight after sampling at 4°C (in 4% Paraformaldehyde) until further use at the laboratory (Figures 1E,F).

### Molecular Identification of Re-crustin

The nucleotide sequence of the Re-crustin was obtained by RT-PCR using 2 μg of template cDNA, with the forward primer 5′- GACAAACACCTCCTCCTCCTCCA−3′ designed from the incomplete 5′ coding sequence of a crustin sequence available in GenBank (accessing number FJ573157) and the oligo (dT)18 primer.

#### cDNA Synthesis

Whole animals were ground in Trizol ReagentTM using the Ultra-Turrax T25^®^ (IKA). RNA was extracted according to the manufacturer's instructions. The concentration of extracted RNA was estimated with the Qubit^®^ 3.0 Fluorometer (ThermoFisher Scientific). RNA extracts were treated by RQ1 RNase-free DNase (Promega) and used for cDNA synthesis with the RevertAid M-MuLV RT kit (ThermoFisher Scientific) according to the manufacturer's protocol. Reaction mixtures for PCR amplifications contained 0.1 μM of each primer, 0.25 mM of each desoxynucleotide triphosphate, 5 × Go Taq G2 Flexi buffer (Promega), and 5 U of GoTaq G2 Flexi DNA polymerase (Promega). The PCR program involved an initial denaturation step at 95°C for 3 min, followed by 39 cycles of 95°C for 1.5 min, 55°C for 1.5 min, and 72°C for 1.5 min, with a final elongation step at 72°C for 5 min.

#### Molecular Cloning and Sequence Analysis

PCR products from each replicate were pooled and then purified with the NucleoSpin^®^ Gel and PCR Clean-up kit (Macherey-Nagel). Purified PCR products were cloned using the TOPO-TA kit (Invitrogen, Carlsbad, CA, USA). Clones were sequenced according to the Sanger method (50) on a 310 ABI prism (Applied Biosystems). Sequences were imported into Geneious version 8.1 software (Biomatters, available from http://www.geneious.com/). Prediction of signal peptide was performed with the SignalP 4.1 program (51) and the presence of conserved domains was tested using the SMART 7.0 protein analysis tool (52). Homology searches were performed using BLAST from NCBI. Multiple alignments of the deduced amino acid sequences (Type I, Type IIa, and Type IIb crustins) were generated using the MAFFT software (scoring matrix BLOSUM62) (53). Maximum likelihood phylogenetic analyses were generated in MEGA X (54) using best-fit WAG model assuming gamma distribution with invariant sites (G+I) for substitution rates. Gaps and missing data were included in data subset as relevant phylogenetic sites. Trees were resampled 1,000 times.

### Determination of the Level and Site of Re-crustin Gene Expression by RT-qPCR

Tissues from branchiostegite, scaphognathite, gills, abdomen, stomach, hepatopancreas and eggs dissected aboard as wells as whole adults and juveniles were ground in Trizol ReagentTM using FastPrep-24^®^ 5G (MP Biomedicals). Total RNA extraction and RT were performed as described in the previous section. The primers used for the quantitative PCR were designed with the Primer3 Input software (http://bioinfo.ut.ee/primer3-0.4.0/primer3/primer3/; www.cgi).

- crustin primers: forward: 5′-ACTGCTGTGAGAACGGGAAC-3′; reverse: 5′-AACATGTTTGAGGGGGTCCT-3′
- Rpl8 primers: forward: 5′-GAAGCTCCCATCAGGTGCCAAGAA-3′; reverse 5′-TTGTTACCACCACCGTGAGGATGC-3′.

The Rpl8 gene was used as the reference gene (55). Real-time quantitative PCR reactions (RT-qPCR) were conducted on a LightCycler^®^ 480 system (Roche) using a hot start enzyme. RT-qPCR assays were submitted to an initial denaturation step of 10 min at 94°C followed by 40 cycles of denaturation at 94°C for 15 s, annealing at 59°C for 1 min and extension at 72°C for 30 s. Reference and target were amplified in separated wells. After amplifications, a melting curve analysis was performed in order to confirm the specificity of the PCR products. Re-crustin primers generated a single and discrete peak in the dissociation curve (data not shown). A negative control and a 5-fold dilution series protocol of pooled cDNAs were included in each run. The 5-fold dilution series were used to construct a relative standard curve to determine the PCR efficiencies and for further quantification analysis. In all experiments, all primer pairs gave amplification efficiencies of 90–100%. Each reaction was run in triplicates. Analysis of relative gene expression data was performed using the ΔΔCq method (56). For each couple of primers, a plot of the log cDNA dilution vs. ΔCq was generated to validate the RT-qPCR experiments (data not shown).

### Immuno-Location of Re-crustin Protein by Western Blot and by Immunohistochemistry

#### Polyclonal Antiserum

The chemically synthesized region of Re-crustin (PTRFGGPPQTCSSDSSCTNNYTDK) was coupled to ovalbumin and used for the immunization procedure of two New Zealand White rabbits (Saprophyte Pathogen free) according to the protocol of Covalab™ (France).

#### Protein Extraction and Electrophoresis

Total proteins were isolated from the samples used for RNA extraction according to the manufacturer's instructions (Trizol ReagentTM, Invitrogen). The white band (interphase) containing the proteins was washed with a solution of 0.3 M guanidine hydrochloride in 95% ethanol, and then resuspended in 9.5 M urea and 2% CHAPS. The protein concentration was determined by the Bradford method using BSA as a standard (57). Proteins were separated by a denaturing SDS-PAGE electrophoresis. The running gel was composed of 12% acrylamide (12% acrylamide; Tris-HCL 1.5 M, pH 8.8; 0.1% SDS; 0.1% ammonium persulfate; 0.01% TEMED) and the stacking gel was composed of 4% acrylamide (4% acrylamide; Tris-HCL 0.5 M pH 6.8; 0.1% SDS; 0.1% ammonium persulfate; 0.01% TEMED). A total of 22 μg of protein was loaded in Laemmli buffer (Tris 125 mM pH 6.8; 20% glycerol; 4% SDS and 5% β-mercaptoethanol). Gels were run at 70 V for 15 min and then at 180 V for 20 min.

#### Immunoblot

The proteins of the SDS-PAGE gel were transferred to a nitrocellulose membrane 0.2 μm (BIO RAD) by semi-dry electro blotting (0.8–1.2 mA/cm^2^). After transfer, the gel was stained by Coomassie Brilliant Blue R-250 (BioRad). The membrane was blocked for 1 h in PBS at 0.1 M containing 0.05% Tween 20 and 5% casein and was then probed with the rabbit polyclonal anti-Re-crustin antibody (1:300 dilution) in the blocking solution (PBS at 0.1 M with 5% w/v non-fat dry milk) overnight at 4°C. After three washes with PBS/0.05%-Tween 20, the membrane was incubated for 1 h in the blocking solution at room temperature with the peroxidase-conjugated anti-rabbit secondary antibody Abcam (1:5000 in PBS at 0.1 M containing 0.05% Tween 20; at 1 h). A Clarity™ Western ECL Substrate (Bio Rad) was used for the chemoluminescence visualization of the immunolabeling with a Kodak Bio Max light film.

#### Immunocytochemistry and Immunohistochemistry

Eggs, juveniles and tissues were fixed aboard in 4% paraformaldehyde. Later, immunohistochemistry was performed on paraffin sections of eggs (thickness of 4 μm), juveniles and adult tissues (thickness of 7 μm). Consecutive paraffin sections were made with a LEICA RM 2255 microtome. Immunocytochemistry and immunohistochemistry were performed with the rabbit anti-Re-crustin (1:400) and the FITC-conjugated anti-rabbit secondary antibody (1:100; Jackson Immunoresearch Laboratories). Samples were examined using a confocal microscope (Zeiss LSM LSM780) and the Fluorescence microscope (Zeiss Axio Imager 2).

### Determination of Antibacterial Activities

#### Bacteria

One Gram-positive *Micrococcus luteus* and one Gram-negative *Vibrio diabolicus* were chosen for being easily carriable and cultivable onboard a ship. *M. luteus* routinely used in laboratory, is found in soil, dust, water and air and *V. diabolicus* was isolated from deep sea hydrothermal vents (58).

#### Samples

Branchiostergites and scaphognathites were crushed with the rotor CoolPrep, MP system (3 times 20 s at 60 rpm) in 0.1 M PBS at 4°C. 10 μL of samples were incubated without (control) or with 0.5 μl of the anti-Re-crustin antibody (dilution 1:400) at 4°C for 20 min.

#### Radial Diffusion Assay

10 μL of each sample were spotted onto LB-agar (Luria-Bertani) plates containing alive *M. luteus* or alive *V. diabolicus* (1 × 10^5^ Colony Forming Unit (CFU)/mL of LB agar). After an overnight incubation at 37°C, the activity was quantified by measuring the diameter of the bacterial growth inhibition.

Experiments were performed in triplicate, once aboard the *Pourquoi pas?* R/V during the BICOSE2 2018 cruise with freshly dissected tissues and twice back to the laboratory in Lille with tissues frozen during the same cruise.

## Results

### Re-crustin, a Novel Member of Type IIa Crustins and a Novel AMP From an Extremophile Organism

**Figure 2:**
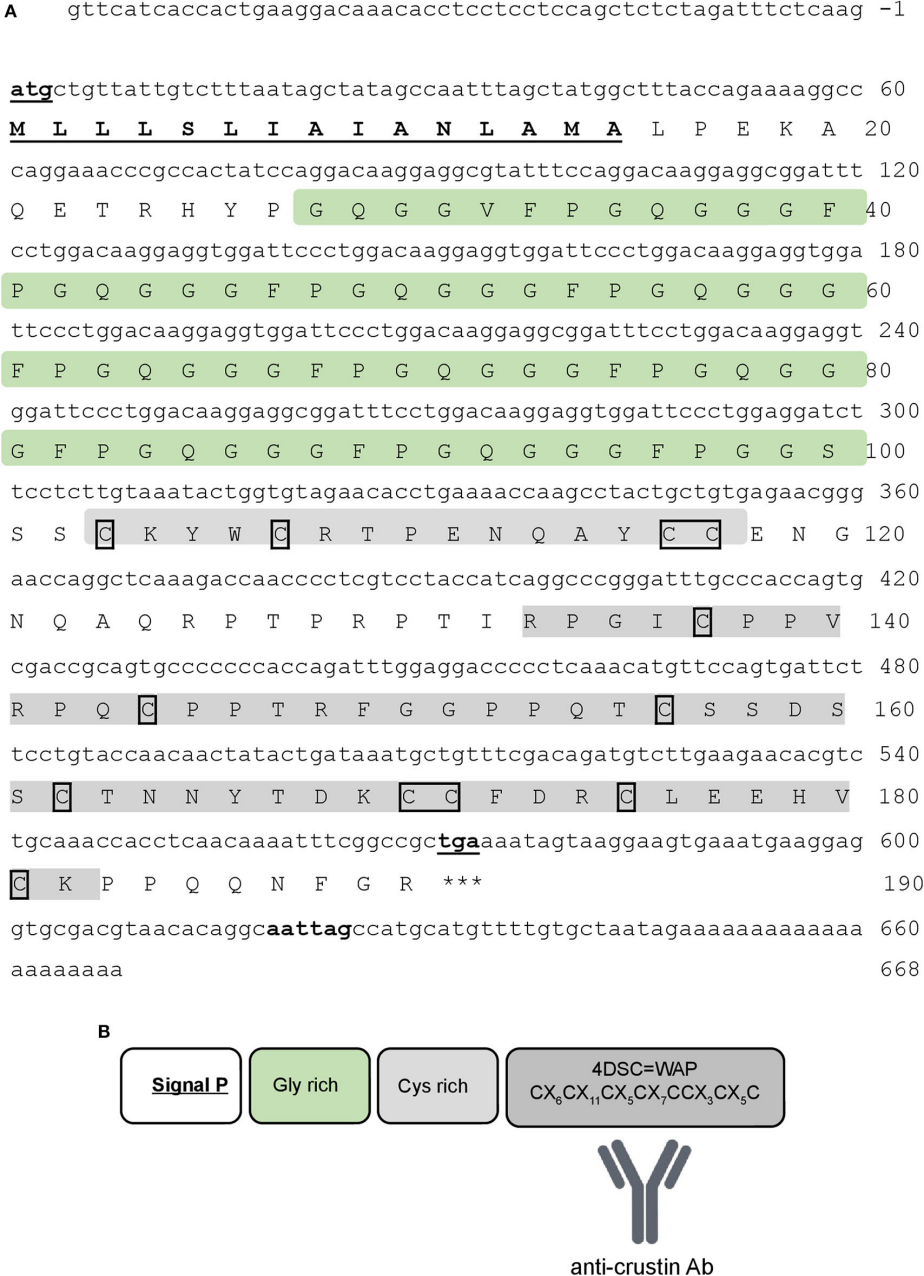
Re-crustin sequence. **(A)** The full-length nucleotide (above) and predicted amino acid (below) sequences of Re-crustin cDNA from *Rimicaris exoculata*. The start and stop codons and the putative polyadenylation site are in bold and underlined. The signal peptide is underlined. The 12 conserved cysteine residues are framed. **(B)** The predicted organization of WAP domain is shown in the dark gray box.

The complete nucleotide sequence of Re-crustin was obtained by 5′-RACE RT-PCR from total RNA extracted from the entire shrimp *R. exoculata* (GenBank accession number: MT102281). Only one sequence of crustin was identified from our molecular subcloning and sequencing. The complete cDNA sequence encodes a precursor of 190 amino acid residues, which includes a 15-residue signal peptide (Figure 2). The mature polypeptide is predicted to consist of 175 residues with a calculated molecular weight of 17.82 kDa and a theoretical isoelectric point (pI) of about 8.5. The mature Re-crustin is composed by a hydrophobic glycine-rich region followed by a C-terminus containing a cysteine-rich region (with 4 conserved cysteine residues) and a single WAP domain (Figure 2B). The glycine-rich region of Re-crustin possesses ten sequential repeats of the heptapeptide Gly-Gly-(Gly/Val)-Phe-Pro-Gly-Gln [GG(G/V)FPGQ].

**Figure 3:**
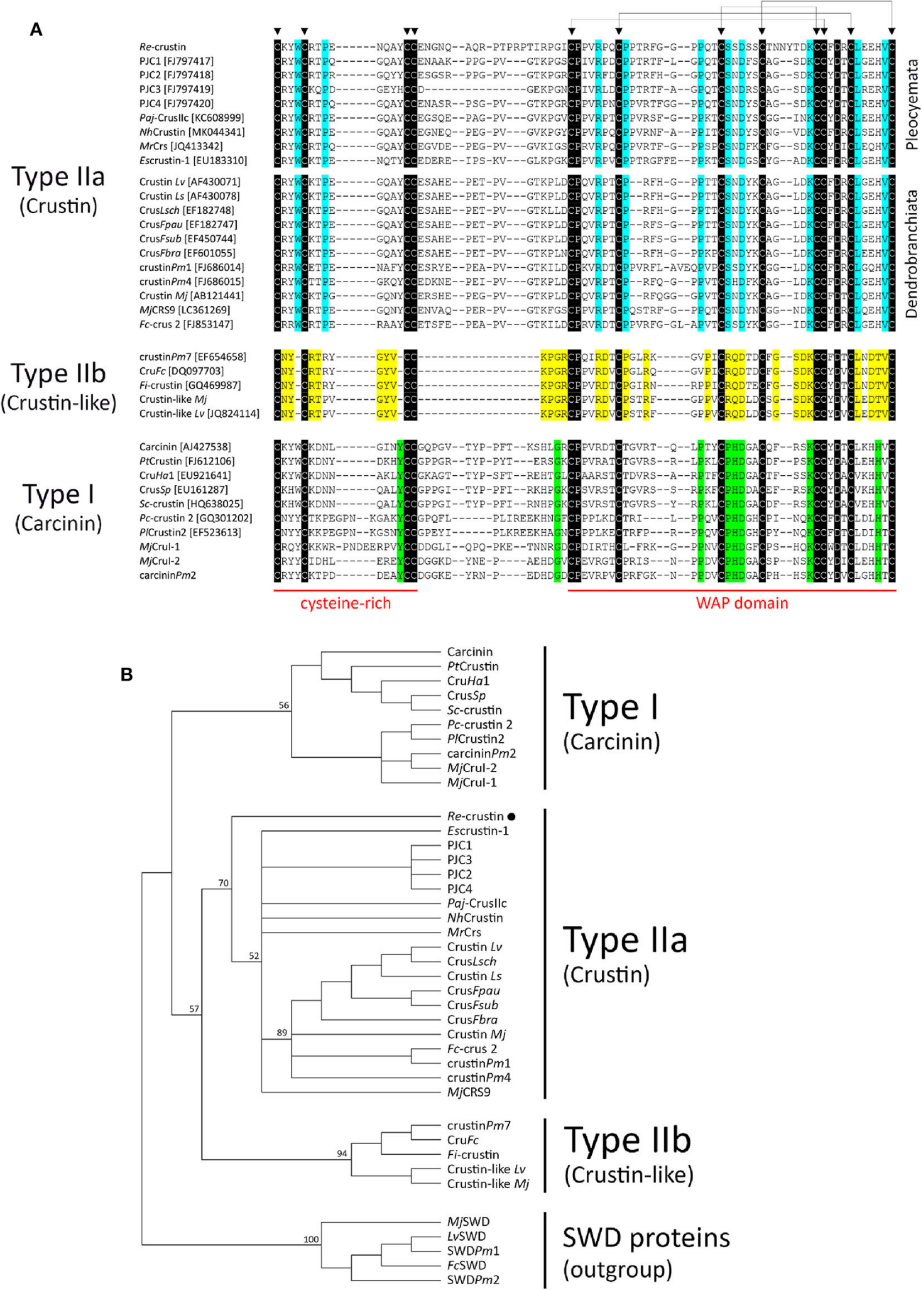
Comparison of Re-crustin with other Type I and Type II crustins from decapod crustaceans. **(A)** Amino acid sequence alignments of the cysteine-rich region and the WAP domain of crustins. Identical amino acid residues are highlighted in black while specific amino acid residues found in Type IIa (“Crustin”), Type IIb (“Crustin-like”), and Type I (“Carcinin”) peptides are highlighted in blue, yellow and green, respectively. Triangles (▼) indicate the 12 conserved cysteine residues. **(B)** Phylogenetic analysis of Type I and Type II crustins. The tree was constructed using the Maximum Likelihood method with bootstrap values calculated from 1,000 trees.

**Figure 4:**
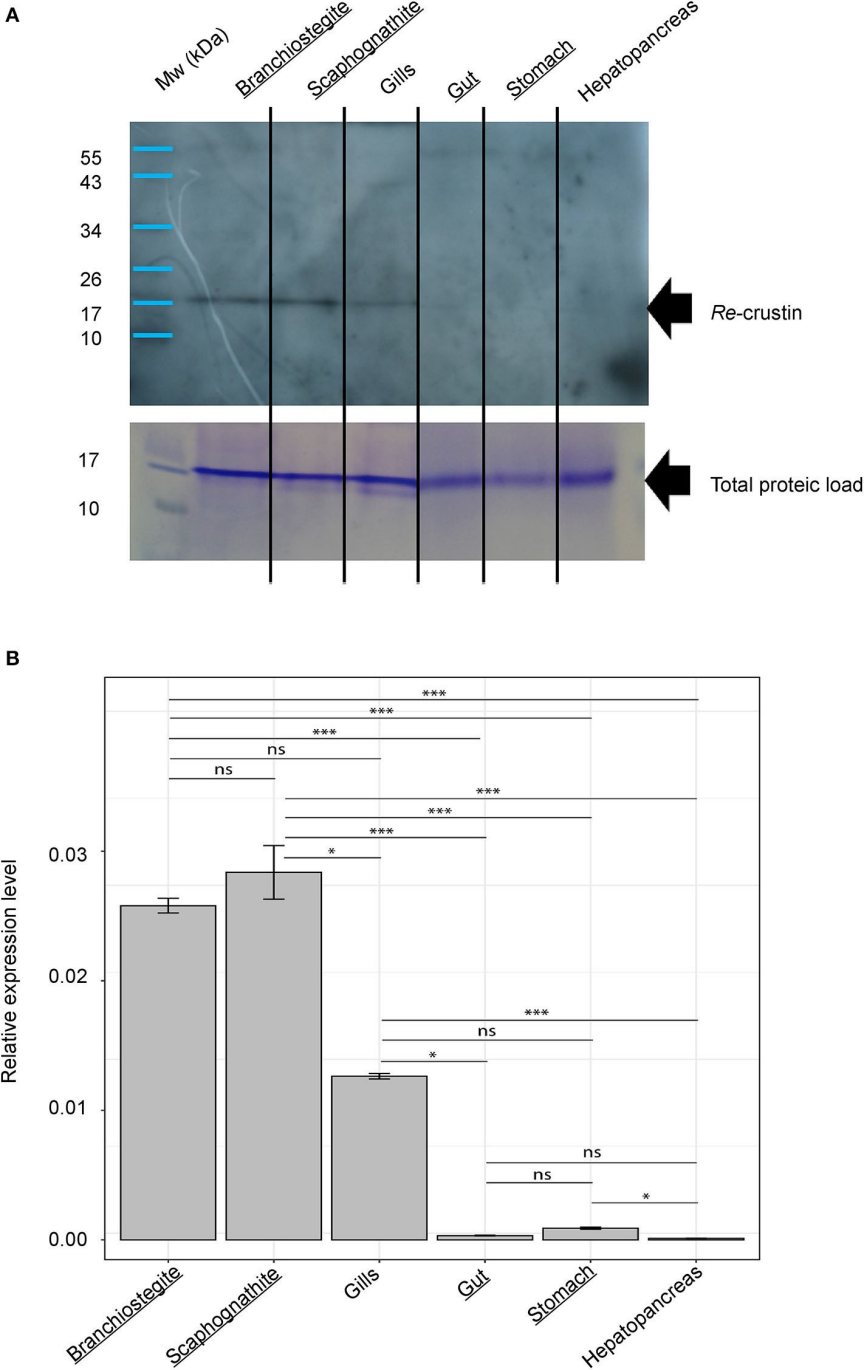
Re-crustin distribution in tissues of adults of *Rimicaris exoculata*. **(A)** Western blot analysis was performed using total protein extracts (22 μg) from branchiostegite; scaphognathite; gills; gut; stomach and hepatopancreas dissected from adults. Immunostaining with the anti-Re-crustin antibody revealed one band of approximately 17 kDa corresponding to Re-crustin mass prediction. Mw, molecular weight markers. Equivalent well-loading was assessed by a generic protein coloration of the gel (Coomassie Brilliant Blue R-250). **(B)** Quantification of the levels of expression of Re-crustin in the cephalothoracic cavity (branchiostegite; scaphognathite; gills) and in the digestive tract (gut; stomach; hepatopancreas) by RT-qPCR analysis using the ΔΔCq method. Known symbiotic tissues are underlined. The graphs show the mean ± SEM for each organ (*n* = 10 in all cases) and significance level for each intergroup comparisons (ns, *p* > 0.05; *p < 0.05; ***p < 0.001; Dunn tests). Kruskal–Wallis: χ2 = 55.812; *p* < 0.001. Reference (Rpl8) and target were amplified in separated wells (*n* > 10 in all cases). A technical triplicate was applied for each sample.

Besides the presence of an N-terminal glycine-rich region, multiple sequence alignment analysis confirmed that Re-crustin is an authentic Type II member from the Sub-Type IIa (Figure 3). The Re-crustin sequence showed highest homology to Type IIa crustins from other decapods from the Pleocyemata suborder, including the red cherry shrimp *Neocaridina heteropoda* (NhCrustin, 67% amino acid identity), the morotoge shrimp *Pandalopsis japonica* (Paj-CrusIIc, 64% amino acid identity), the Japanese spiny lobster *Panulirus japonicus* (PJC1-4, 59–66% amino acid identity) and the Chinese mitten crab *Eriocheir sinensis* (Escrustin-1, 63% amino acid identity). Within Type II crustins from penaeid shrimp (Dendrobranchiata), Re-crustin was 52–59% identical to Type IIa crustins and 44–53% identical to Type IIb crustins. On the other hand, the mature Re-crustin displayed 36–44% identity to Type I crustins from decapods from both Pleocyemata and Dendrobranchiata suborders. Less than 30% amino acid identity was observed between Re-crustin and Type III (single WAP domain-containing proteins or SWD) and Type IV crustins (double WAP domain-containing proteins or DWD). Western blot analysis from total protein extracts showed a band at the predicted molecular weight confirming (i) the specificity of the antibody designed to recognize the WAP domain of Re-crustin and (ii) the translation of the Re-crustin transcripts (Figure 4B).

### In Adults, Re-crustin Is Produced by Tissues on Which Ectosymbionts Develop

**Figure 5:**
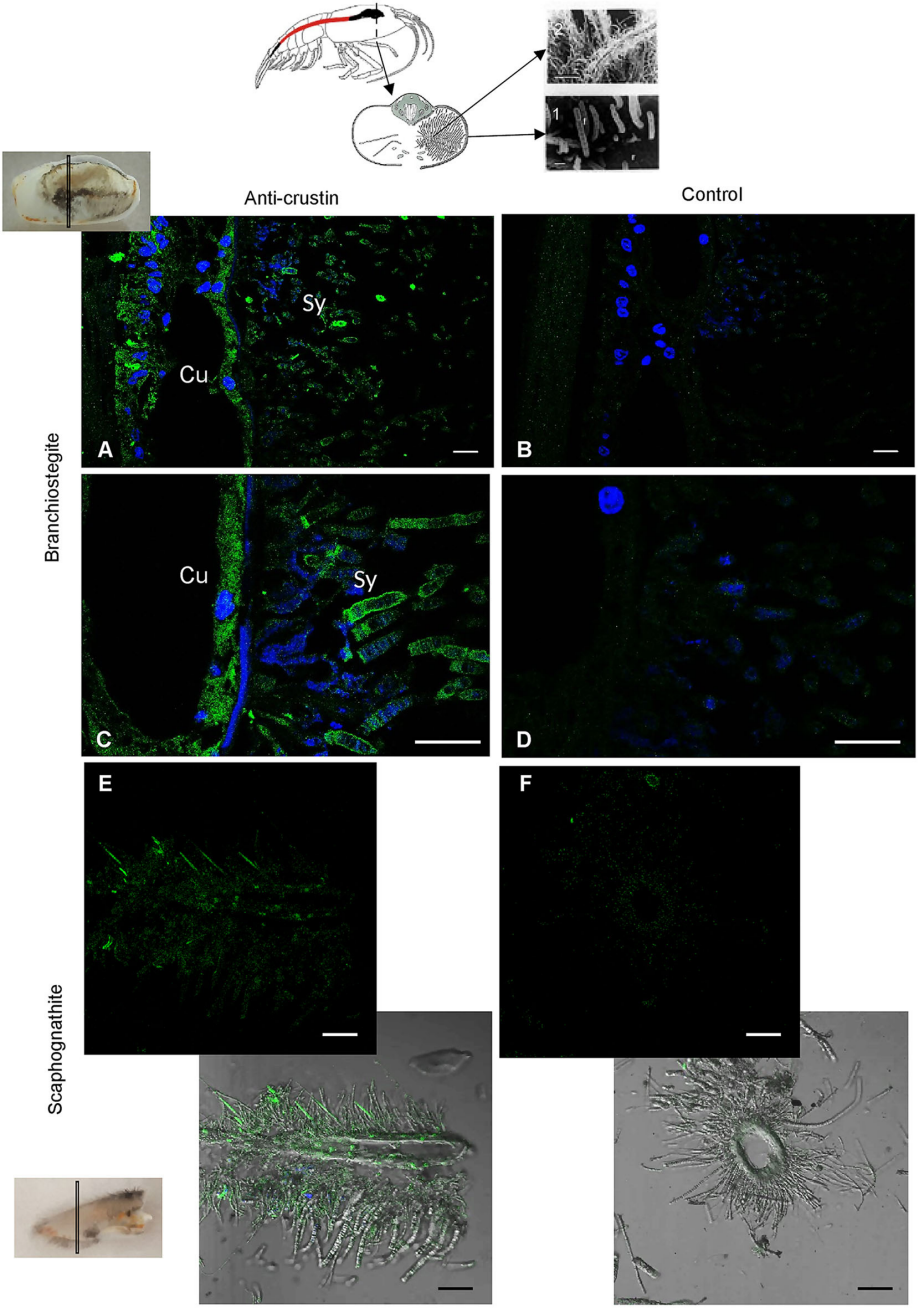
Immunolabeling on paraffin sections with a thickness of 7 μm of Re-crustin on pieces of carapace colonized by bacteria. Schematic *Rimicaris exoculata* cephalothoracic chamber illustrated by two photos in electron microscopy (1, 2) the filamentous *Campylobacteria* epibionts. **(A–D)** Branchiostegite and **(E,F)** scaphognathite. **(A–C)** The anti-crustin antibody specifically labels in green the tissue that lines the inside of the cephalothoracic cavity and also covers the surface of some attached bacteria. **(E)** Some bacteria attached to the scaphognathite are also labeled. **(B–F)** No labeling appears with the pre-immune control. Nucleic acids are labeled in blue (DAPI). The white field is superimposed in E and F to see the shadows of the structures. The observations were made using the confocal microscope, Zeiss LSM 780. Scale bars correspond to 20 μm. Sy, symbiont; Cu, Cuticle.

RT-qPCR and Western blot analyses were performed on exactly the same tissues from the same individuals (Figure 4). Results showed the presence of both transcripts (Figure 4A) and proteins (Figure 4B) in the pieces of the cephalothoracic cavity. Neither the transcripts nor the proteins were detected in the gut, the hepatopancreas and the stomach. The major Re-crustin producing tissues were clearly those on which the ectosymbiotic community of the cephalothoracic cavity develops, i.e., the branchiostegites and scaphognathites. Cellular localization of Re-crustin was then investigated in these pieces of carapace colonized by symbionts using immunohistochemistry and confocal microscopy analyses (Figure 5). Immunolabeling with the anti-crustin antibody provided evidence for the synthesis of Re-crustin by the epithelium cells beneath the cuticle of the branchiostegites (Figure 5A–D) and scaphognathites (Figures 5E,F) from adults and its accumulation on the cuticle that delimits the cephalothoracic cavity. Interestingly, Re-crustin covered some of intact ectosymbiotic bacteria anchored to these mouth pieces (Figures 5C,E).

### Re-crustin Produced by Branchiostegites and Scaphognathites Displays Antibacterial Activities

**Figure 6:**
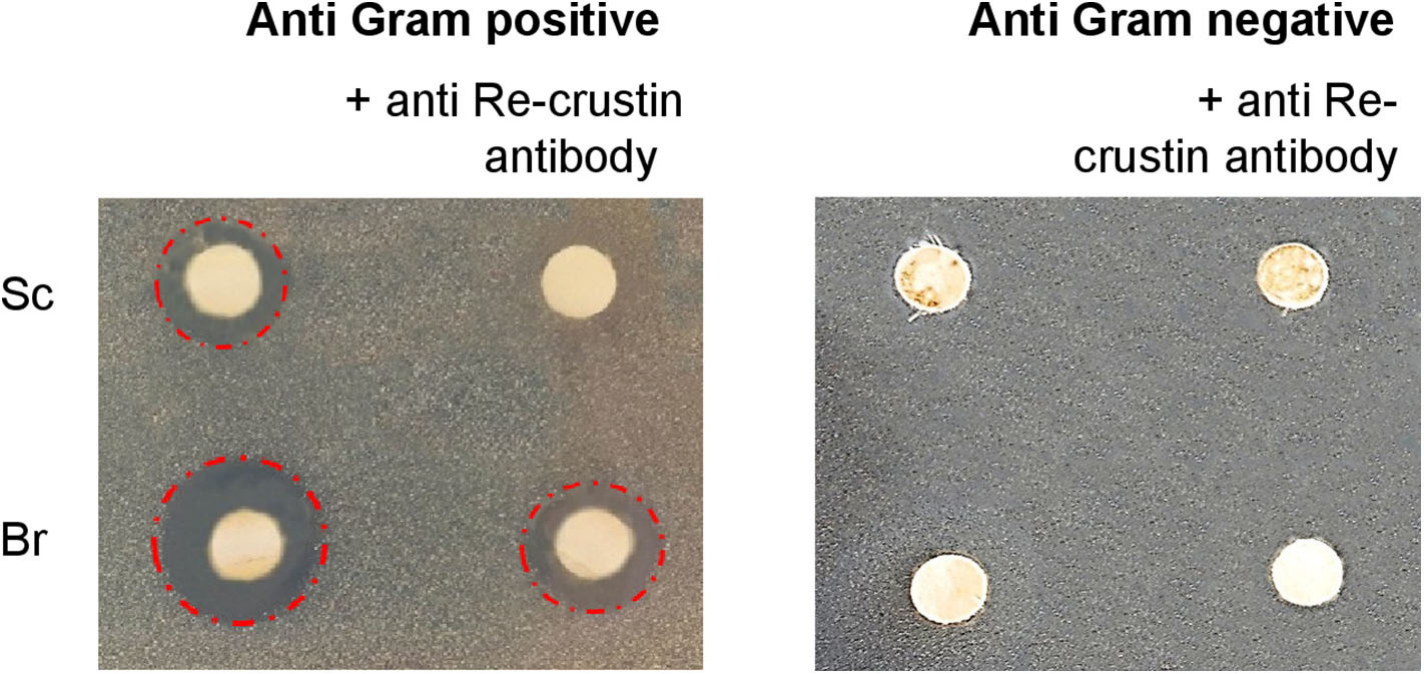
Antimicrobial activities of crude extracts of branchiostegites (Br) and scaphognathites (Sc) against Gram-positive and Gram-negative bacteria. The antibody added to the extracts acts as a blocking agent of the endogenous Re-crustin. The red circles underline the antimicrobial activities.

Antibacterial assays performed in triplicate from crude extracts of branchiostegites and scaphognathites showed anti-bacterial activities against the Gram-positive *M. luteus* but not against the Gram-negative *Vibrio diabolicus* (Figure 6). Part of this antibacterial effect is significantly reduced when the endogenous Re-crustin is blocked by adding the specific Re-crustin antibody to the extract, confirming the production and the antibacterial activity of Re-crustin in both scaphognathites and branchiostegites. Since the anti-Re-crustin antibody does not fully inhibit the antibacterial activities, active substances others than Re-crustin are presumably produced by the scaphognathites and the branchiostegites.

### The Production Site of Re-crustin Along the Life Cycle of the Shrimp Is Correlated With the Acquisition of Ectosymbionts

**Figure 7:**
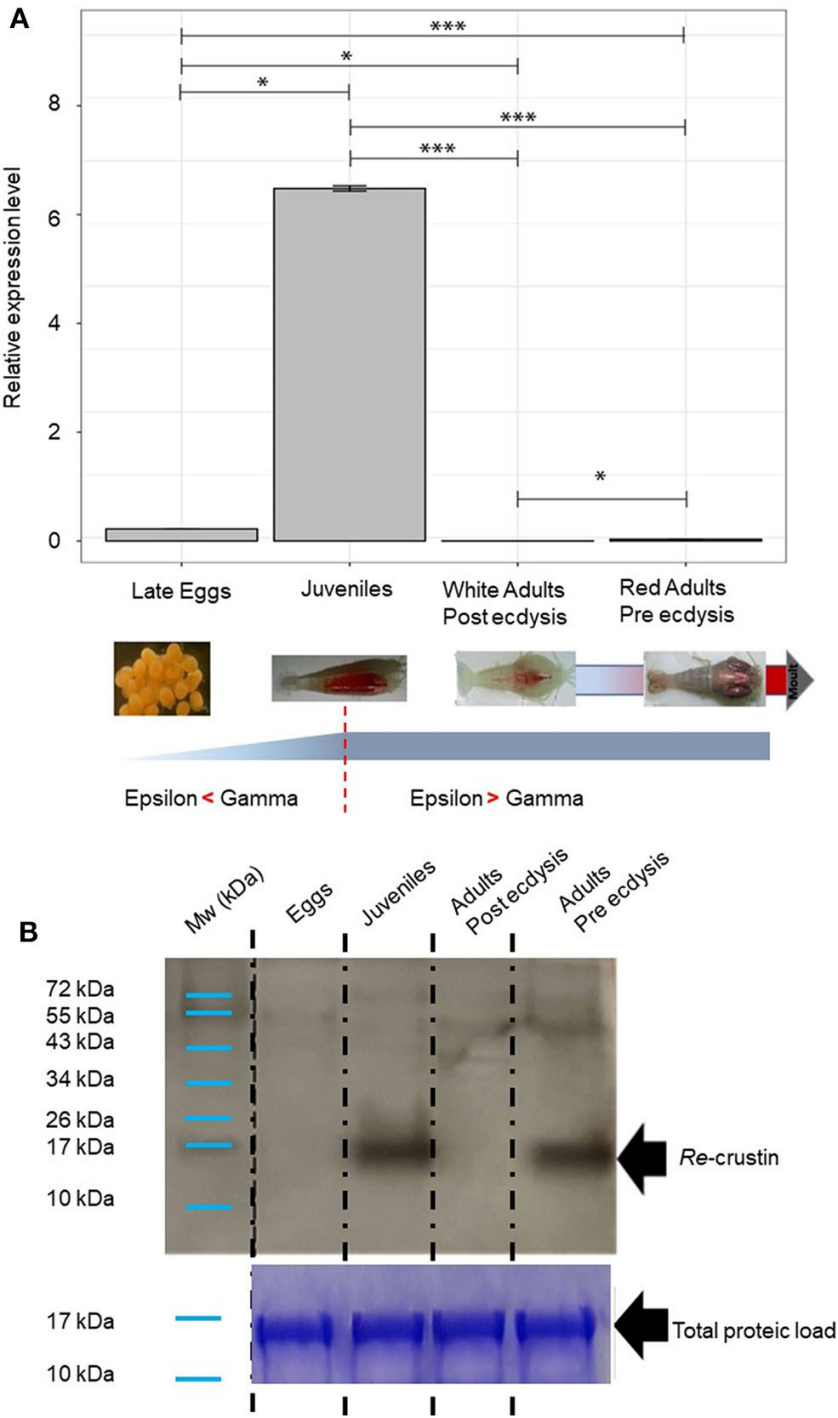
Re-crustin distribution along the *Rimicaris exoculata* life cycle and molt stages. As represented, a shift occurs in symbiotic population between Gammaproteobacteria and Campylobacteria along the host life stage with the first one dominating at early stages while the second dominating at adult stages (5, 47) for early stages and (4, 33) for adults. **(A)** Gene expression analysis by RT-qPCR. Reference (Rpl8) and targets were amplified in separated wells (*n* > 10 in all cases). The graph shows the mean + SEM for each life stages and molt stages (*n* = 10 in all cases) as well as significance level for each intergroup comparison (ns, *p* > 0.05; *p < 0.05; ***p < 0.001; Dunn tests). Kruskal–Wallis: χ2 = 36.596; *p* < 0.001. Reference (Rpl8) and targets were amplified in separated wells. A technical triplicate was applied for each sample. **(B)** Western blot analysis was performed using total protein extracts (22 μg) from eggs, juveniles, adults (beginning of molt cycle), and adults (end of molt cycle). Immunostaining with the anti-Re-crustin antibody revealed one band of approximately 17 kDa corresponding to Re-crustin mass prediction. Mw, molecular weight markers. Equivalent well-loading was assessed by a generic protein staining of the gel (Coomassie Brilliant Blue R-250).

**Figure 8:**
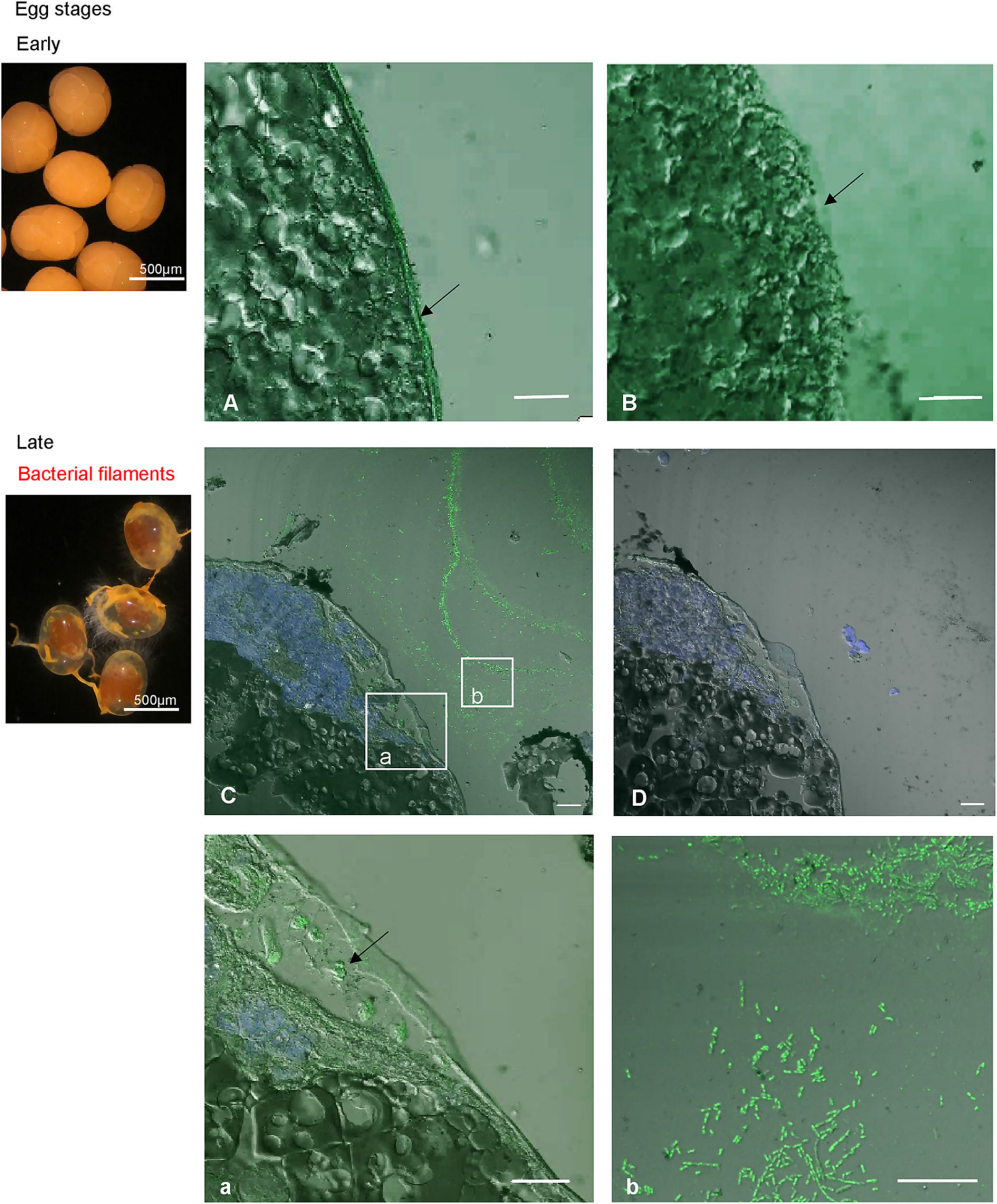
Immunolabeling on paraffin sections with a thickness of 4 μm of Re-crustin on eggs of early stages (**A,B**, no biofilm) and of late stages (**C,D**, a,b, orange biofilm). A slight labeling is visible in the panels corresponding to eggs at young stage, with the specific antibody **(A)** and is absent with the pre-immune control **(B)**. On late stages, the biofilm that covers the eggs is clearly labeled (**C**, b for a closer view), and some vesicles containing Re-crustin are visible beneath the membrane of the egg (**C**; a; for a closer view). **(D)** No labeling is visible with the pre-immune control. Nucleic acids are labeled in blue (DAPI). The white field is superimposed to see the shadows of the structures. The observations were made using the confocal microscope, Zeiss LSM 780. Scale bars correspond to 20 μm.

**Figure 9:**
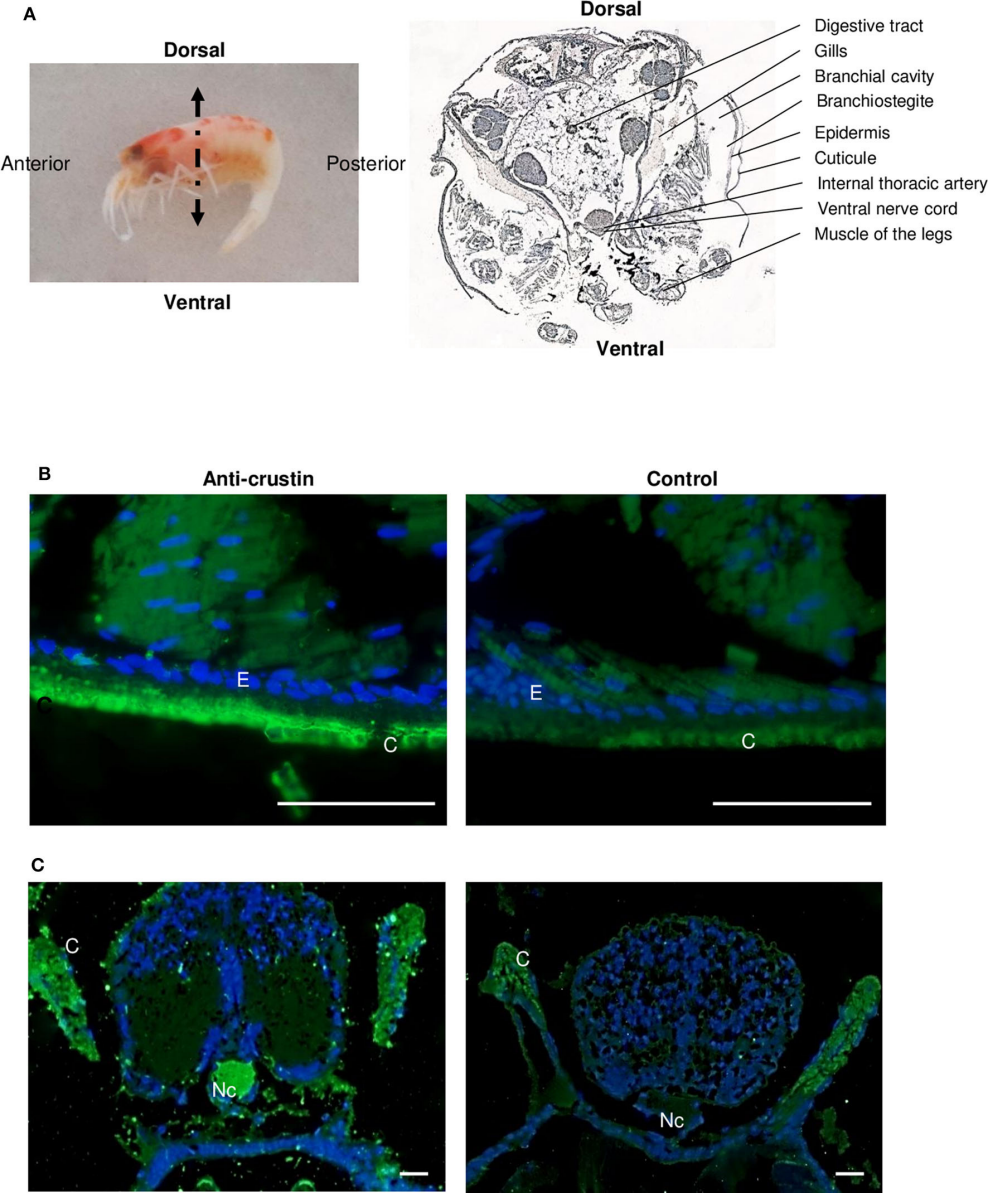
Immunolabeling on paraffin sections with a thickness of 7 μm of Re-crustin on juveniles. **(A)** Image of a juvenile fixed in 4% paraformaldehyde to illustrate the detailed section shown on the right. The anti-crustin antibody specifically labels in green the cuticle **(B)**, gills and nerve cord **(C)** in juveniles. No labeling is visible in the control panels. Nucleic acids are labeled in blue (DAPI). The observations were made using the fluorescence microscope, Zeiss Axio Imager 2. Scale bars correspond to 20 μm. Abbreviations used: C, cuticle; E, Epidermis; G, Gills; Nc, nerve cord.

The transcriptomic and protein levels of Re-crustin were investigated at different stages of the shrimp life-cycle for which the colonization states by the ectosymbionts were already described (Figure 7) (5, 47). In late eggs, when they are covered by large amount of symbionts (47), the gene is slightly expressed (Figure 7A), but the protein is not detected in the western blot analysis (Figure 7B). Using immunohistochemistry, which is a more sensitive method, we detect a small amount of the protein in the membrane of freshly spawned eggs (early eggs, almost deprived of symbionts, Methou et al. (47) (Figures 8A,B). In late stages, Re-crustin was immunodetected into vesicles beneath the cell membrane (Figures 8CA,D) and on the bacteria that form the biofilm surrounding the eggs (Figures 8CB,D). Juvenile specimens are young shrimps recruited close to adults aggregates where they have been sampled, and start their development toward adult symbiotic life (Methou et al., submitted). RT-qPCR data using total RNAs extracted from these whole juveniles combined with western blot show that Re-crustin transcripts and proteins are highly abundant at this transition stage (Figure 7A). Re-crustin was immuno-localized on the cuticle that delimits the cephalothoracic cavity, in the gills and also in the nervous system (Figure 9).

**Figure 10:**
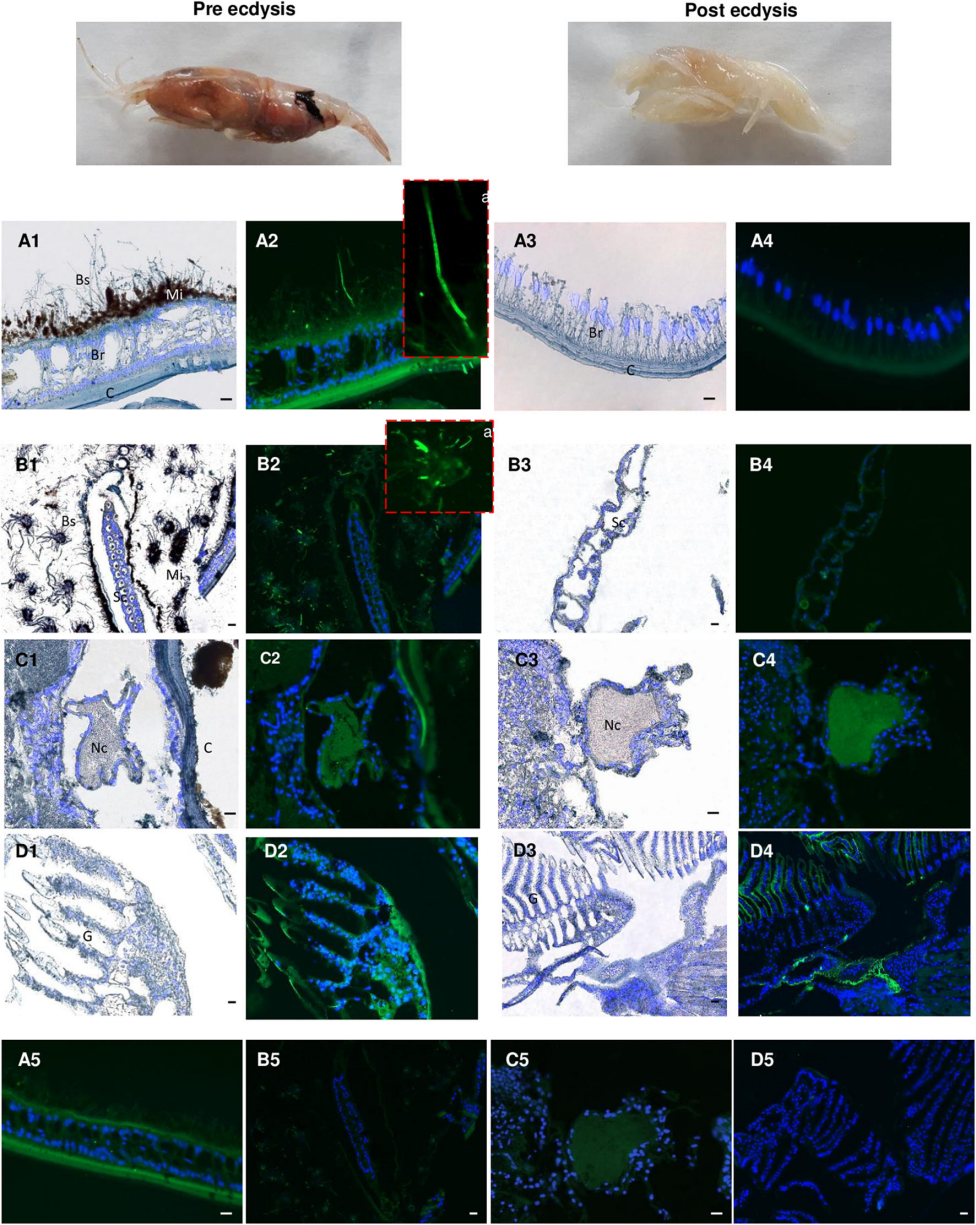
Immunolabeling on paraffin sections with a thickness of 7 μm of Re-crustin in adult shrimps before and after molting. Two images present an adult shrimp at the end of the molting cycle (Pre ecdysis) and after molting (Post ecdysis). **(A2-a)** The anti-crustin antibody specifically labels in green the tissue that lines the inside of the cephalothoracic cavity and also covers the surface of some attached bacteria at the Pre ecdysis but not at the Post-ecdysis stage **(A4)** which is devoid of symbionts. **(B2-a)** Some bacteria attached to the scaphognathites are labeled at Pre ecdysis. No labeling is observed in the scaphognathites free of ectosymbionts of postecdysial adults **(B4)**. An immune-staining of the Re-crustin is observed in the Nerve cord **(C2-C4)** and in the gills **(D2-D4)** of adults at both the pre and post ecdysis stages. Nucleic acids are labeled in blue (DAPI). **(A5-B5-C5,D5)** No labeling appears with the pre-immune control. The white field is shown in **(A1, A3, B1, B3, C1, C3, D1, D3)** to see the structures. The observations were made using the fluorescence microscope, Zeiss Axio Imager 2. The 20 μm scale bars are placed on the white fields and on the pre-immune controls. Abbreviations used: Br, branchiostegites; C, cuticle; Bs, Bacterial symbionts; G, gills; Mi, mineral deposits; Sc, scaphognathite; Nc, nerve cord.

Because the life of adults is punctuated by molt cycles, the same protocol was applied to “white” adults at the beginning of their molt cycle, where almost all epibiont have been eliminated, in comparison with “red” adults at the end of their molt cycle, highly colonized but where epibionts are encrusted in minerals impairing their activities (39) (Figure 10). In both cases, Re-crustin transcripts were not detected by RT-qPCR using RNA extracted from whole animals, probably because of an over dilution of the transcripts (Figure 7A). By contrast, the protein was abundantly present in red adults at their pre-ecdysial stage while it was undetectable in adults that have just molted and are starting a new cycle (Figure 7B). Immunohistochemistry showed an accumulation of Re-crustin in the epidermis beneath the cuticle colonized by epibionts of red animals only (Figure 10).

## Discussion

We open hypothesis for a novel biological role for gene-encoded antimicrobial host defense peptides (AMPs) in crustacean-microbe interactions. Our results revealed that the expression of a new member of the classic crustin AMP family (Re-crustin) is spatio-temporally correlated with the establishment of the ectosymbiotic microbial communities inhabiting the cephalothoracic cavity of the extremophile deep-sea shrimp *R. exoculata*.

Different from its Pleocyemata counterparts that mainly produce Type I crustins, *R. exoculata* expresses in its tissues a N-terminal glycine-rich crustin that belongs to the Sub-Type IIa. Indeed, the Type II comprises a distinct group of crustins usually found in penaeid shrimp (Dendrobranchiata suborder) that is subdivided into two Sub-Types: Type IIa (“Crustins”) and Type IIb (“Crustin-like”) (44). Although unusual, the presence of Type IIa crustins has been reported for other decapod crustaceans from the Pleocyemata suborder (59, 60). On the other hand, Type IIb crustins were only described in penaeid shrimp species (46).

The presence of a signal peptide in the Re-crustin precursor, together with the results obtained from the western blot analysis, provides evidence of its processing prior to the release of the Re-crustin into the extracellular compartment where it exerts its biological properties. The accumulation of Re-crustin on the surface of some ectosymbionts, as evidenced by immunohistochemistry, also supports the extracellular secretion and clearly shows an interaction of Re-crustin with the ectosymbiotic community of the cephalothoracic cavity *in vivo*. This interspecific interaction appears as an important function of Re-crustin in *R. exoculata*. Our multiple approaches all demonstrate that Re-crustin is essentially produced by the mouthparts in the cephalothoracic cavity, which is hugely colonized by the ectosymbionts (4, 5, 30, 32). Re-crustin was slightly detected in the digestive tract, which is colonized by a microbial community different from the one of the cephalothoracic cavity (28, 29). This suggests a low contribution of Re-crustin in this organ and underlines the importance of the molecule in the cephalothoracic cavity of *Rimicaris*. When tested for their production of antibiotics, the scaphognathites and branchiostegites showed an antibacterial activity that was partially, but not only, due to Re-crustin, thus suggesting the synthesis of other still undiscovered antimicrobial substances by these appendages or their associated bacteria. No activity was observed against the tested proteobacteria, confirming Re-crustin as belonging to the Type IIa crustins which are known to be mainly active against Gram-positives (61) while the Type IIb crustin from *Penaeus monodon* (crustinPm7/Crus-likePm) showed antibacterial activity against both Gram-positive and Gram-negative bacteria (62, 63). Because they are also uncultivable, the ectosymbionts could not be used for *in vitro* antimicrobial assays. However, immunodetection with the anti-Re-crustin antibody showed that the AMP covers the surface of the filamentous ectosymbionts (mainly composed of Gram-negative Proteobacteria/Campylobacterota) without killing them. A control of the bacterial growth via bacteriostatic activities of Re-crustin as observed in the endosymbiostasis of beetles cannot be excluded (64). One could also hypothesize that when embedding the ectosymbionts, Re-crustin contributes to their success by acting as an anti-competitive agent against other environmental bacteria and by favoring indirectly or directly their growth. A chemoattractant effect of Re-crustin favoring the recruitment of target symbionts and their subsequent attachment to the host might also be proposed as an explanation to the accumulation of Re-crustin at the surface of some ectosymbionts. The production of the recombinant molecule is planned to enlarge the spectrum of antibacterial activities and to decipher the other putative biological functions of Re-crustin.

Re-crustin is probably part of a cocktail of AMPs and immune receptors, such as the recently characterized type C lectin (10), acting synergistically to shape the symbiont community and to prevent the colonization of the gills by pathogens and/or competitors such as Gram-positive bacteria and also Archaea. Further investigations will have to be performed to identify the other antibiotics involved in the emblematic ectosymbiotic association of *Rimicaris*.

Re-crustin was also immunodetected in the central nervous system (CNS). Further investigations should be performed to determine whether the expression sites are the neurons themselves or the epithelial cells infiltrated into the nervous system as documented for the crustin named PET-15 in the spiny lobster (65). A multifunction of Re-crustin appears also as a potential hypothesis to explore, following e.g., results on the neuronal growth activities of AMPs produced by the CNS of other invertebrates (66).

To go further into the role of Re-crustin in the ectosymbiostasis of *Rimicaris*, we explored the spatio-temporal correlations between the levels of production of Re-crustin and the ectosymbiotic acquisition/loss/re-colonization events that punctuate the life cycle of the hydrothermal shrimp (Figure 1). Our data show that Re-crustin, is already present at the surface of just spawned eggs. The synthesis is enhanced in late eggs still attached to the abdomen of the mother, as they are being colonized by a dense mat of bacteria. Interestingly, Re-crustin secreted by late eggs covers the bacteria forming the biofilm. The biofilm formation appears concomitant to Re-crustin synthesis/secretion in eggs during their embryonic stages and thus may contribute to the development of the immune system. Re-crustin may also serve as an anti-competitive and/or a growth factor for the symbionts. During their embryonic development, *R. exoculata* egg envelopes are colonized by bacterial communities that partly differ from adult's symbiotic communities for late stage eggs (5, 47). After hatching, larvae undergo several molting events, still uncharacterized, to the juvenile stage A, while dispersing in the water column before their recruitment to a new hydrothermal vent field (5, 67). Although we cannot rule out the possibility that some of the bacteria present on eggs during embryonic development are carried along during post-hatching larval life until recruitment, recruited juveniles appear to be mostly colonized anew shortly after settlement by an ectosymbiotic community i.e., the one of the cephalothoracic cavity identified in surrounding adults (40). In late eggs and even much more pronounced in juveniles recruited to the sites inhabited by adults, the peak of synthesis of Re-crustin likely corresponds to the development of a novel bacterial community corresponding to the adult one. Re-crustin would then appear as a developmental, metamorphic signal induced by the symbiotic community associated to the eggs and to the juveniles starting their development toward adult symbiotic forms.

Immunohistochemistry combined with RT-qPCR and western blot data also show a spatial correlation between the AMP and the symbionts, with an abundant presence of Re-crustin in eggs and in the epidermis cells beneath the cuticle of the branchiostegites and scaphognathites carrying the ectosymbionts, in recruited juveniles and in adults.

As far as we know, only few studies have been devoted to the investigation of crustin expression during the early stages of crustacean's life and none were correlated with the associated bacterial community (43, 45, 68, 69). Hauton and colleagues have shown that Type I crustin expression levels are similar in lobster *Homarus gammarus* postlarvae stages IV and VI (68). Larvae of the shrimp *P. monodon* have been reported to express a Type IIb crustin transcript at high levels at all stages of development from nauplii IV through to juveniles (46). In all reported cases, an immune function of crustins in larvae was proposed but their involvement in the symbiostasis was not investigated.

Like most crustaceans and contrary to metabole insects, *Rimicaris* still molt during their adult stage. Molting results in the complete renewal of the cuticle (including mouth appendages) together with the loss of the attached ectosymbiotic community, notably on scaphognathites and branchiostegites in our case. Interestingly, the production of Re-crustin by the scaphognathites and branchiostegites reaches a peak when the re-colonization occurs, suggesting a role of Re-crustin in the control of the symbiosis acquisition and presumably in the selection and success of the appropriate bacteria from the habitat. Surprisingly, the protein load is increased in the pre ecdysis stage while the mRNA expression of Re-crustin is not. In addition to the technical explanation (see results), a neosynthesis might also occur in between the two stages (i.e., in between the post and the pre ecdysis) and should be investigated. An increased gene expression starting after the post ecdysis would lead to an accumulation along the molt cycle of the protein (embedding the symbionts that have colonized the gill chamber) which quantity would reach a maximal at the pre-ecdysis stage whereas the mRNA transcription would have already stopped. Since *Rimicaris* forms large and dense colonies constituted by individuals desynchronized in their molting and development, the symbiotic bacteria remain always available within the population, such as on shed exuvia, thus allowing the horizontal transfer of the symbionts. Because Re-crustin accumulates on the surface of both the symbionts and the cuticle of the appendages, the molecule may also serve as a communication pathway (such as a chemoattractant effect see before) in between the host and the bacteria thus favoring the recruitment and the horizontal transfer of the free-living symbionts.

During the extremely short molt cycle (10 days), the symbiont growth is accompanied by a progressive mineral accumulation, caused in part by the symbiont activities (39, 70). In pre-ecdysial advanced stage, the mineral crust completely surrounds the symbionts and some lysis forms can even be observed. The recovery induced by molting could “stifle” the symbionts that would no longer be able to properly feed their host. The host might thus trigger its molt to recycle its ectosymbionts. Therefore, the *R. exoculata* molt cycle could be compared to the concept of symbiotic trade-off retrieved in insects (64) whereby insects offer “board and lodging” to the endosymbionts as long as they can rely on their metabolic supply, and recycle them by autophagy and apoptosis when the cost of symbiont maintenance overcomes the provided benefits.

Our results overall highlight a novel AMP sequence from an extremophile organism and suggest the role of AMPs in the establishment of vital ectosymbioses that take place along the life cycle of marine organisms. *R. exoculata* enlarged cephalothoracic cavity offers a mechanic but also, an immune protective and growth environment for the symbionts against the harsh hydrothermal vent conditions and their microbial competitors while the symbionts provide nutrients *via* a transcuticular transfer (6) and maybe an immunity to the shrimp. The developmental and metamorphic role of the ectosymbiosis *via* the production of Re-crustin would have to be investigated to decipher the correlated production of the AMP with the symbiotic colonization and the transition stages of the host.

## Data Availability Statement

The datasets generated for this study can be found in the Re crustin, GenBank accession number: MT102281.

## Author Contributions

AT, M-AC-B, and FP designed the research. SL, CB-W, VC-G, LD, and PM developed reagents and performed experiments. SL, CB-W, RR, VC-H, M-AC-B, and AT analyzed the data. SL, CB-W, RR, VC-H, PM, FP, M-AC-B, and AT wrote the manuscript. All authors contributed to the article and approved the submitted version.

## Conflict of Interest

The authors declare that the research was conducted in the absence of any commercial or financial relationships that could be construed as a potential conflict of interest.
